# How do the results of the RADIANT trials impact on the management of NET patients? A systematic review of published studies

**DOI:** 10.18632/oncotarget.8601

**Published:** 2016-04-05

**Authors:** Sara Pusceddu, Filippo De Braud, Giuseppe Lo Russo, Laura Concas, Daniela Femia, Claudio Vernieri, Alice Indini, Barbara Formisano, Roberto Buzzoni

**Affiliations:** ^1^ Medical Oncology Department, ENETs Center of Excellence, Milan, Italy; ^2^ Fondazione IRCCS Istituto Nazionale dei Tumori, Milan, Italy

**Keywords:** everolimus, neuroendocrine tumors

## Abstract

In the last five years, everolimus has demonstrated efficacy in the treatment of neuroendocrine tumors (NETs) of different origins; its efficacy and safety were explored in the RADIANT trials, the last of which (RADIANT-4) has been recently published (December 2015). Overall, evidence collected from the RADIANT studies holds promise to change clinical practice for the treatment of NETs.

In this paper, we comment on the role of everolimus within the therapeutic algorithm for NETs treatment, based on the systematic analysis of the RADIANT trials and our experience.

## INTRODUCTION

The incidence of neuroendocrine tumors (NETs) is increasing worldwide [1]. NETs represent a heterogeneous class of neoplasms, with tumor behavior - and therefore patient survival - depending upon several factors including primary site, tumor histology, proliferative index, and staging [2]. The management of NETs is challenging, and only few therapeutic options are currently available. In recent years, targeted therapies such as sunitinib and everolimus have been tested for the treatment of NETs, with overall satisfactorily results [3].

The phosphatidylinositol 3-kinase (PI3K)/AKT/mammalian target of rapamycin (mTOR) pathway plays a central role in the regulation of cell proliferation, apoptosis, cell cycle, metabolism, and angiogenesis [4]. Of note, a number of studies have suggested that the PI3K/AKT/mTOR signaling pathway is strongly implicated in the pathogenesis and progression of neuroendocrine tumors [4–9]. Therefore, a targeted therapy able to directly inhibit the mTOR pathway, e.g. everolimus, does represent a particularly suitable strategy for the treatment of NETs.

In the last five years, everolimus has demonstrated activity in the treatment of NETs of different origins; its efficacy and safety were explored in the RADIANT trials, the last of which (RADIANT-4) was published in December 2015 [10–13]. Overall, evidence collected from the RADIANT studies holds promise to change clinical practice in the treatment of NETs.

In this paper, we provide our personal view on how do the results of the RADIANT studies impact on the management of NET patients, and we comment on the role of everolimus within the therapeutic algorithm for NETs treatment on the basis of the systematic analysis of the RADIANT trials and our experience.

## EVIDENCE COLLECTION

Papers for consideration in this manuscript were collected based on a PubMed search, using pertinent keywords (‘RADIANT’ AND ‘Neuroendocrine tumors’ AND everolimus). No limitations in terms of language or publication date were applied. The first search was last updated on December 10^th^ 2015, and it was then supplemented by manually browsing the reference section of identified papers; studies included to authors' personal collection of literature were considered as well. A supplementary research was conducted at the revision of the paper (March 2016).

Papers were then selected for inclusion on the basis of their relevance for the topic, as judged by the authors.

## THE RADIANT STUDIES

### RADIANT 1

The rationale for the RADIANT 1 study was based upon the results of a preliminary Phase II study, in which patients with advanced low-intermediate carcinoid or islet cells NETs received everolimus 5 mg/day (*n* = 30) or 10 mg/day (*n* = 30) and octreotide LAR 30 mg every 28 days [5]. Overall, the response rate (RR) was 20% at the intent-to-treat analysis; at the per protocol analysis, there were 13 partial responses (PRs) (22%) and 42 stable diseases (SDs; 70%). Median progression-free survival (PFS) was 60 weeks, and the 3-year survival rate was 78%. Main grade 3/4 toxicities were hypophosphatemia (11%), fatigue (11%), and diarrhea (11%).

The above-mentioned findings paved the way for the international RADIANT 1 study. This open-label, Phase II trial evaluated the efficacy and safety of everolimus in patients with metastatic pancreatic NET (pNET) who progressed on chemotherapy [10]. Patients were stratified by prior octreotide therapy (stratum 1: everolimus 10 mg/day, *n* = 115; stratum 2: everolimus 10 mg/day plus octreotide long-acting release [LAR], *n* = 45). At central analysis, 11 PRs (9.6%) and 78 SDs (67.8%) were reported in stratum 1; median PFS was 9.7 months. In stratum 2, there were two PRs (4.4%), while 36 patients (80%) achieved SD; progression of disease (PD) was never observed. In this group, median PFS was 16.7 months. The authors of the RADIANT 1 trial concluded that daily everolimus, either with or without concomitant octreotide LAR, showed promising antitumor activity and was well tolerated in patients with advanced pNETs after failure of prior systemic chemotherapy.

### RADIANT 2

In the international randomized, double-blind, placebo controlled phase III RADIANT 2 trial, the combination of everolimus (10 mg/day) plus octreotide LAR was compared with octreotide LAR alone in 429 patients with low-grade or intermediate-grade NET associated with carcinoid syndrome [11]. The primary endpoint was PFS; its median values were 16.4 months in the everolimus plus octreotide LAR group and 11.3 months in octreotide LAR-only group, respectively (hazard ratio [HR] 0.77, 95% CI 0.59-1.00). In line with the results of the RADIANT 1 study, most drug-related adverse events were of grade 1 or 2; most frequent adverse events (all grades) included stomatitis (62% *vs* 14%), rash (37% *vs* 12%), fatigue (31% *vs* 23%), and diarrhea (27% *vs* 16%).

Of note, three major imbalances should be taken into account when considering the findings of the RADIANT 2 trial. First, WHO performance status was less favorable in the everolimus arm compared with the placebo arm. Second, there was a higher incidence of pulmonary primary tumors in the everolimus arm (15% *vs* 5%). Third, patients assigned to the combination arm had a more frequent prior used of chemotherapy. Also due - at least in part - to those imbalances, this study did not reach statistical significance with respect to PFS; statistical significance was pre-specified to be set at 0.0246, and it was actually 0.026.

On the other hand, the results of a number of subgroup analyses of the RADIANT 2 lend support to the efficacy of everolimus. In a sub-analysis including only patients with lung NET (everolimus plus octreotide LAR, *n* = 33; octreotide LAR only, *n* = 11), median PFS was 2.4-fold longer in the combination arm (13.6 *vs* 5.6 months), with a 28% reduction in the risk of progression (HR, 0.72; 95% CI 0.31-1.68) [14]. More patients on everolimus plus octreotide LAR (67%) experienced tumor shrinkage than those receiving octreotide LAR only (27%). The safety profile was consistent with the results of the core trial. The authors of this sub-analysis suggested that the advantage for everolimus+octreotide LAR over octreotide LAR alone was clinically-relevant, and therefore supported the continued evaluation of everolimus in patients with lung NETs. Overall, similar findings were reported in the subgroup analysis of patients with colorectal NETs [15]. In this analysis, patients assigned to the combination arm (*n* = 19) experienced a significantly longer median PFS (29.9 months) than those on octreotide LAR only (6.6 months; *n* = 20). Everolimus plus octreotide LAR also determined a significant reduction in the risk of disease progression (HR: 0.34; 95% CI 0.13-0.89; *p* = 0.011). Although no objective responses were observed, tumor shrinkage was more frequently reported with combination treatment (67% *vs* 37%). Last, another recently published sub-analysis of the RADIANT 2 trial evaluated the impact of previous treatment with somatostatin analogue (SSA) on the safety and efficacy of everolimus [16]. In total, 339 patients enrolled in the trial were previously exposed to SSAs. Overall, more patients on previous SSA therapy had a history of flushing symptoms (77%), diarrhea (86%), or both (63%) compared with SSA-naive subjects (62%, 62%, and 24%, respectively). Of note, patients in the combination arm showed longer median PFS regardless of previous SSA exposure (with: PFS 14.3 months, 95% CI 12.0-20.1; without: 25.2 months, 95% CI, 12.0-NR) than those on octreotide LAR only (with: 11.1 months, 95% CI 8.4-14.6; without: 13.6 months, 95% CI 8.2-22.7).

### RADIANT 3

The international randomized, placebo-controlled, phase III RADIANT 3 trial was the largest study ever conducted in the setting of NETs [12]. In this trial, 410 patients with advanced, low-grade or intermediate-grade pNET who showed radiologic progression within the previous 12 months before enrolment were randomly assigned to receive everolimus (10 mg/day; *n* = 207) or placebo (*n* = 203), both associated with best supportive care. The primary endpoint was PFS. If radiologic progression occurred during the study, patients assigned to placebo were offered open-label everolimus. Median PFS was significantly longer in the everolimus group, compared with controls. In more details, it was 11.0 months with everolimus and 4.6 months with placebo, with a 65% reduced risk of progression or death (HR 0.35; 95% CI 0.27-0.45;*p* < 0.001). The estimated proportion of patients progression-free at 18 months were 34% (95% CI 26-43) with everolimus and 9% (4-16) with placebo. This PFS was consistent at the subgroup analysis, regardless of age, gender, ethnicity, exposure to SSA, performance status, tumor differentiation. These findings are of utmost clinical relevance, since they suggest that everolimus may represent an effective treatment option in all patients with well-differentiated or moderately-differentiated pNET, without any parameter suggesting exclusion from treatment.

Overall survival (OS) did not differ between patients treated with everolimus or those assigned to placebo (HR, 1.05; 95% CI 0.71-1.55). This lack of advantage can probably attributed to the high proportion of patients (73%) who were crossed over from placebo to everolimus.

In line with other RADIANT trials, adverse events were mostly of grade 1 or 2 severity; however, some grade 3 or 4 events were more frequent with everolimus, including anemia (6% *vs* 0%) and hyperglycemia (5% *vs* 2%).

In a recent sub-analysis of the RADIANT 3 trial, the impact of previous chemotherapy on the efficacy everolimus was evaluated [17]. In total, 204 (50%) out of the 410 enrolled in the trial were chemo-naive. Everolimus prolonged PFS regardless of prior chemotherapy (prior chemotherapy group: 11.0 months with everolimus and 3.2 months with placebo; HR 0.34; 95% CI 0.25-0.48;*p* < 0.0001; chemo-naive group: 11.4 *vs* 5.4 months; HR 0.42; 95% CI 0.29-0.60; *p* < 0.0001).

### RADIANT 4

The RADIANT-4 trial is a prospective, multicenter, randomized, double-blind, placebo-controlled, phase III study on patients with well-differentiated (G1 or G2) advanced NET of gastrointestinal (GI) or lung origin [13]. All patients had no history of and no active symptoms related to carcinoid syndrome, and had reported radiologic progression in the last 6 months. Enrolled subjects were randomly assigned, in a 2:1 ratio, to receive either everolimus 10 mg/day (*n* = 205) or placebo (*n* = 97) plus best supportive care. The primary endpoint was PFS.

In total, 175 patients had GI NET and 90 had lung disease. At central review analysis, everolimus-treated patients showed a prolonged median PFS, as compared with those receiving placebo (11.0 *vs* 3.9 months, HR 0.48; 95% CI 0.35-0.67; *p* < 0.00001). Overall similar results were observed at investigator assessment (14.0 *vs* 5.5 months; HR 0.39; 95% CI 0.28-0.54; *p* < 0.00001). This benefit in PFS was consistent at all subgroup analyses: in particular, there was a 50% improvement in PFS for patients with lung tumors and a 44% benefit for those with GI NETs. Tumor shrinkage was observed in 64% of patients assigned to the everolimus group and 26% of those in the placebo group. The objective response rate was 2% in the everolimus group and 1% in the placebo arm; on the other hand, disease control rate was 82.4% with everolimus and 64.9% with placebo. Death rates in the everolimus and placebo group were similar, although the everolimus group was treated longer. The first pre-planned interim OS analysis suggested that everolimus might be associated with a reduction in the risk of death (HR 0.64; 95% CI 0.40-1.05; *p* = 0037, whereas the boundary for statistical significance was 0.0002) compared with placebo. Adverse events were consistent with the safety profile for everolimus.

## DISCUSSION

As a whole, the RADIANT studies showed that the introduction of everolimus in the pharmacological armamentarium for the treatment of NETs represents a key step forward in the therapy of this heterogeneous group of diseases. In fact, the efficacy and safety of everolimus have been consistently shown in well-differentiated NETs from all origins. Given the favorable results obtained in the pivotal studies, everolimus has been approved for the treatment of advanced pNETs and its approval procedure in GI and lung carcinoids is ongoing. However how this “pan-availability” of everolimus will influence clinical practice?

In pNETs, a number of therapeutic options such as sunitinib, SSAs, chemotherapy and peptide receptor radionuclide therapy (PRRT) are also available, while other agents - e.g. pazopanib - showed promising, although preliminary, results [18, 19]. At present, everolimus undoubtedly represents one of the most effective therapy options in this subset of patients, but its role within the therapeutic sequence (upfront therapy or after disease progression on chemotherapy/SSA) remains unclear. The subgroup analysis of the RADIANT 3 study showed the similar efficacy of everolimus in patients pretreated with chemotherapy and in those naïve for treatment [17]. On the other hand, the results of the recent phase II ITMO study, by Bajetta et al, suggest higher response rate and prolonged PFS with upfront everolimus combined with octreotide LAR, compared with administration at a later line of treatment [20]. Although the limited size and the different study design do not allow the comparison between the ITMO study and the RADIANT 3 trial, the study by Bajetta et al suggests further investigating the optimal therapeutic sequence for pNETs. Moreover, there is a need to find clinical and/or biological criteria to identify patients who can gain most benefit from upfront everolimus and those who may receive this molecule at progression from first-line treatment.

At present, given the lack of head-to-head trials (the multicentric SEQTOR trial is still open for enrolment; NCT02246127), we believe that given its antiproliferative efficacy and tolerability profile everolimus can represent an ideal upfront treatment in G2 pNET patients, with rapidly-evolving disease and high disease burden.

On the other hand, everolimus may represent an ideal second-line treatment for G1 pNET patients with indolent disease and low tumor burden after progression on SSA, in line with the results of the PROMID and CLARINET trials [21, 22]; these patients often present a long life expectancy and therefore tolerability and quality of life become crucial in selection of treatment.

**Figure 1:**
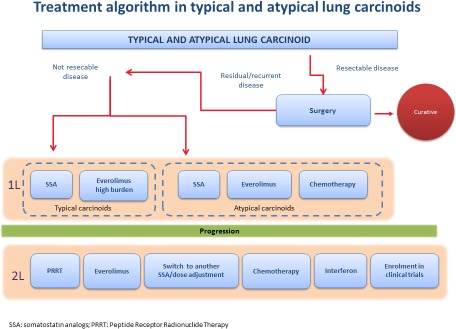

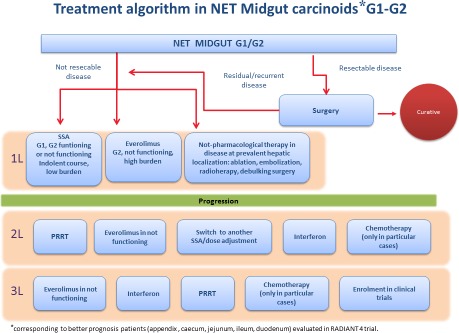
Treatment algorithm – proposed by the authors – for the treatment of lung carcinoids (Panel A) and of midgut G1-2 carcinoids (Panel B) after the publication of the RADIANT-4 trial

With respect to GI and lung carcinoids, the RADIANT 4 study will likely have a major impact on clinical practice, especially for lung NETs (Figure 1). Indeed, it was the first randomized study to specifically show that everolimus is significantly effective in patients with lung tumors. We believe that this study represent a major breakthrough, because, to date, there has not been any properly established treatment option for lung carcinoids. In particular, we think that everolimus may be particularly suitable for patients with more aggressive and rapidly progressing disease such as those with atypical carcinoids, in whom upfront treatment can be suggested. Moreover, data on second-line therapy with everolimus are even more grounded, also compared with those available for chemotherapy and PRRT, which are mostly derived from retrospective series or not-randomized studies in a mixed population of patients with typical carcinoids and subjects with atypical carcinoids.

With respect to GI carcinoids, we believe that, although the RADIANT 4 study showed promising results their impact on clinical practice will be less remarkable than for lung NETs. In fact, the RADIANT 4 trial demonstrated the significant prolongation of PFS in patients with well-differentiated, advanced, progressive, nonfunctional NET regardless of lung or GI origin, as well as in site subgroups. However, the preplanned HR analysis by stratification factors of central review shows that when patients with better prognosis (appendix, caecum, jejunum, ileum, duodenum, and NET of unknown primary) are compared with those at worse prognosis (lung, stomach, rectum, and colon except caecum), a better HR for PFS was observed for patients with worse prognosis (0.43) and in those with moderately-differentiated NETs G2 patients (0.49). HR in the “better prognosis” subgroup was 0.63, and HR in patients with well-differentiated NETs G1 was 0.57. Overall, these findings suggest that worse grade of differentiation and worse prognosis may be associated with higher efficacy of everolimus.

Based on these findings, we believe that the use of everolimus will be somehow limited in the upfront setting for “better prognosis” patients with appendix, caecum, jejunum, ileum, duodenum (midgut) NET, in whom a number of other treatment options such as SSA and PRRT are available. In addition, the approval for everolimus has been sought only for non-functioning patients, thus excluding from treatment functioning patients such as those with midgut carcinoids. Therefore, everolimus may be particularly suitable as upfront therapy or after progression for midgut patients with non-functioning G2 disease in whom SSA or PRRT therapy may be not indicated.

On the other hand, for less indolent GI carcinoids, prospective evidence from the phase III RADIANT 4 is of paramount importance. In fact, with the exception of SSA [22, 23], other available therapeutic options, such as chemotherapy and PRRT, have been investigated only in retrospective or phase II not-randomized studies.

In conclusion, treatment of NETs can actually be quite complex, primarily because they are a highly heterogeneous group of tumors. We believe that 2015 has been a very exciting year for NETs management, because now we have many new studies that have provided us with new treatment options. The RADIANT studies, involving thousands of patients and several research Institutions all over the world, showed exciting results that show the “pan-availability” of everolimus across all NET subtypes likely allowing relevant improvements in patients' care and a modification of therapeutic algorithms. The identification of the optimal treatment sequence(s), correct treatment timing and the selection of patients currently represent the issues to be explored in controlled clinical trials.

At present, a number of studies on the use of everolimus in the treatment of NET patients are ongoing or are awaiting their final results. The results of the RAMSETE trial (NCT00688623) will likely be released in late 2016: this single-arm, multicenter, phase II study evaluates everolimus monotherapy in the treatment of metastatic, non-syndromic NETs. In addition, everolimus is being investigated within different combination regimens with other targeted therapies (e.g., sorafenib [NCT00942682] or bevacizumab [NCT01229943]), temozolomide (NCT00576680 in patients with advanced pNETs and NCT02248012 in patients with advanced G3 gastroenteropancreatic NETs), and even metformin (NCT 02294006). Another intriguing combination therapy is the concomitant treatment with everolimus and pasireotide. This strategy is being assessed the COOPERATE-1 study (NCT01263353), now completed, conducted in patients with advanced metastatic NETs and in the 3-arm, phase II LUNA trial (NCT01563354) which compares everolimus+pasireotide LAR with the corresponding monotherapies in subjects with NET at lung/thymus.
